# Caregivers' perceived adequacy of support in end-stage lung disease: results of a population survey

**DOI:** 10.1186/1471-2466-11-55

**Published:** 2011-11-25

**Authors:** David C Currow, Morag Farquhar, Alicia M Ward, Gregory B Crawford, Amy P Abernethy

**Affiliations:** 1 Discipline, Palliative and Supportive Services, Flinders University, Adelaide, Australia; 2 General Practice & Primary Care Research Unit, Department of Public Health & Primary Care, University of Cambridge, UK; 3 Southern Adelaide Palliative Services, Repatriation General Hospital, Daw Park, South Australia, Australia; 4 Discipline of Medicine, University of Adelaide, Adelaide. South Australia, Australia; 5 Division of Medical Oncology, Department of Medicine, Duke University Medical Centre, Durham, North Carolina, USA

## Abstract

**Background:**

End-stage lung disease (ESLD) is a frequent cause of death. What are the differences in the supports needed by caregivers of individuals with ESLD at end of life versus other life-limiting diagnoses?

**Methods:**

The South Australian Health Omnibus is an annual, random, face-to-face, cross-sectional survey. In 2002, 2003 and 2005-2007, respondents were asked a range of questions about end-of-life care; there were approximately 3000 survey participants annually (participation rate 77.9%). Responses were standardised for the whole population. The families and friends who cared for someone with ESLD were the focus of this analysis. In addition to describing caring, respondents reported additional support that would have been helpful.

**Results:**

Of 1504 deaths reported, 145 (9.6%) were due to ESLD. The ESLD cohort were older than those with other 'expected' causes of death (> 65 years of age; 92.6% versus 70.6%; p < 0.0001) and were less likely to access specialised palliative care services (38.4% versus 61.9%; p < 0.0001). For those with ESLD, the mean caring period was significantly longer at 25 months (standard deviation (SD) 24) than for 'other diagnoses' (15 months; SD 18; p < 0.0001). Domains where additional support would have been useful included *physical care, information provision*, and *emotional and spiritual support*.

**Conclusions:**

Caregiver needs were similar regardless of the underlying diagnosis although access to palliative care specialist services occurred less often for ESLD patients. This was despite significantly longer periods of time for which care was provided.

## Background

Caregivers of people at the end-of-life face emotional, social and financial sequelae. At a population level, there is the potential for poorer health outcomes including morbidity and mortality. Understanding the pressures on caregivers may help to develop interventions that improve these outcomes [1,2].

Quantitative [3-5] and qualitative [6,7] studies of patients with end-stage organ failure have described the needs of this population. The trajectory of end-stage lung disease (ESLD), whether obstructive or restrictive, is one of inexorable decline punctuated by disease exacerbations. For caregivers of ESLD patients, there is uncertainty about the length of time and intensity of care needed [8]. Several mostly qualitative studies have been undertaken to understand better the impact of care when people have chronic obstructive pulmonary disease (COPD) [9-12]. Findings include the impact of providing care on the well being of caregivers [10], a dearth of quality information to support caregivers [13] and poor contingency planning for acute worsening especially of shortness of breath [9]. Although family burden may be high, for many people providing care for family members with COPD is a positive experience [12]. Despite these papers, given the magnitude of ESLD in the community, and the health and social systems' reliance on family and friends to provide care, there is relatively little information about the role of caregivers for people with ESLD or their needs. The research that is available is built almost exclusively around people with COPD and is silent on other causes of ESLD.

A basic description of the caring experience for ESLD patients is needed, especially as it pertains to the most intense and uncertain period in the illness - end-of-life. It is imperative to understand what additional support would be of benefit and, subsequently, how this might improve caregiver outcomes. The aim of this population-based study was to describe differences in caring and the support needed by caregivers of people with advanced ESLD compared to caregivers of people with other life-limiting diagnoses. The null hypothesis was that there were no demonstrated differences between caregivers for people with ESLD and caregivers for people at the end of life with other diagnoses.

## Methods

### Setting

South Australia has a population of 1.56 million people (7% of Australia's population), the majority of whom live in Adelaide (population 1.1 million) with the balance residing in small non-metropolitan centres (populations less than 30, 000) [14].

### Subjects

One interview (60-90 minutes in duration) is conducted per household with the person over age 15 who most recently had a birthday. If this person declines to participate, the person cannot be replaced by another member of the household.

### Study Design

The South Australian Health Omnibus is an annual, face-to-face, cross-sectional survey. A multi-stage, systematic area sampling method is used; annual survey results are standardised against weighted population-based norms to accommodate random imbalances in sampling. Omnibus is run by a commercial research organisation using trained interviewers who survey between September and December annually [15]. The survey processes described here are unchanged since the inception of the survey in 1991 and are not able to be modified at the discretion of individual research teams.

### Sampling Schema

**Figure 1:**
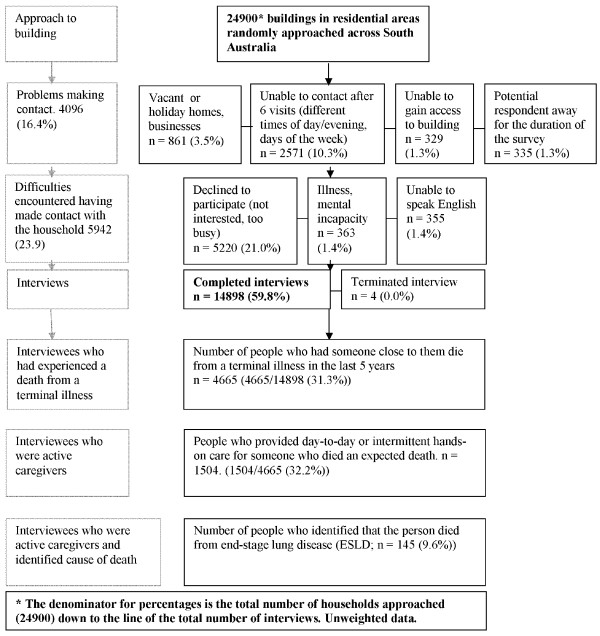
**Flowchart of participation in the South Australian Health Omnibus 2002-3, 2005-7 Participation rate 71.6% (14902/20804)**.

The survey draws 75% of its sample from greater Adelaide. Annually, more than 5, 000 properties are approached seeking a resident to participate (Figure 1). Not all properties are residential as they may include vacant land or businesses. Participation rates are calculated on the number of residences. Where contact cannot be made with a household, a further five attempts are undertaken to contact them at different times of the day and days of the week.

In Australia, the smallest census administrative data area is a collector district (CD), which has approximately 200 dwellings. There are 2, 041 CDs in Adelaide (of which 340 are randomly chosen with an equal probability of selection for each). There are another 1, 010 non-metropolitan CDs of which 100 are included. All eight towns in the state with a population of more than 10, 000 are included and towns with populations 1, 000 to 10, 000 are randomly selected with probability of selection proportional to the size of the town. This gives a statewide total of 440 starting points. A map in each selected CD is numbered for all corners, and a random number generator is used to choose the starting point in each CD. Each surveyor uses a standard algorithm to move from the starting point through the CD.

### Data Quality

Data are double entered. Missing responses are followed up by telephone by a research supervisor. For quality assurance, 10% of each interviewer's respondents are selected randomly and also re-contacted, asked to reconfirm their eligibility and re-answer a sub-set of questions. Aggregated data are then anonymised before release to researchers.

### Data sources - survey tool

Researchers pay to submit their own questions and in return are provided with de-identified demographic data for all respondents and the responses to the researchers' own submitted questions.

### Variables - questions in the Health Omnibus

Approximately 200 questions about health beliefs and behaviors from a wide range of disciplines are included. Annually, prior to the main survey, a pilot of 50 interviews with members of the general public is conducted to test questions, validate the survey instruments, and reconfirm survey procedures.

### Questions of caregivers for people with advanced life-limiting illness

**Figure 2:**
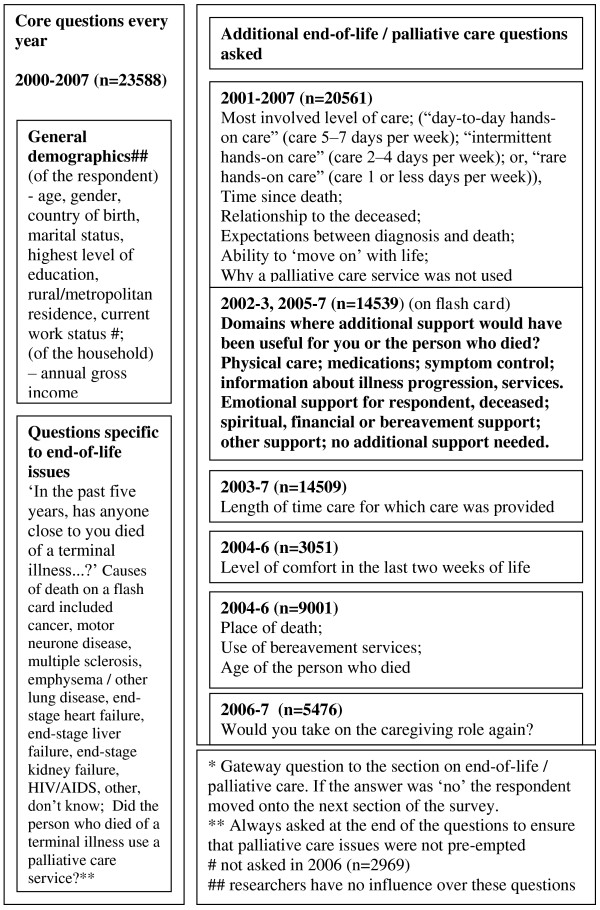
**Questions asked about palliative and end-of-life care in the South Australian Health Omnibus 2000-2007**. __ indicates data used in this analysis.

**Figure 3:**
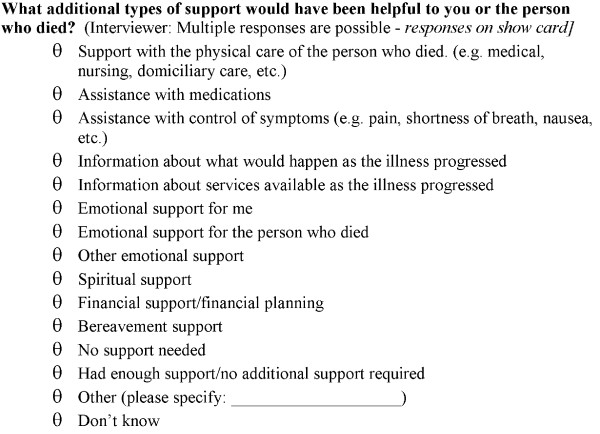
**Questions on unmet needs in the face-to-face interview population-based South Australian Health Omnibus 2002-3, 2005-7**.

For the purposes of this study, a 'gateway' question was asked - 'In the past five years, has anyone close to you died of a terminal illness..?' (Figure 2) If the answer was 'No', then they proceeded to the next domain of questions; if 'Yes', the balance of end-of-life questions was asked. The level of care provided and domains where additional support may have been helpful were asked of the bereaved (Years 2002, 2003 and 2005-2007; Figure 3) [2]. A specific question derived from the literature regarding grief asks respondents to reflect on the process of 'moving on' [16].

### Analysis

Initially, data were weighted by the inverse of the respondent's probability of selection for the survey, adjusted for participation rates in metropolitan and rural areas, and then re-weighted and directly standardised against the South Australian population (2006) for gender, 10 year age group, country of birth and region of residence [2,8]. Before combining the dataset for the five years using appropriate direct standardisation macros, each yearly dataset was compared to exclude significant demographic differences. Each respondent was assigned a standardised weight and only weighted data analysed.

Demographic information collected in the survey included the respondent, limited information about the deceased care recipient, their relationship and the nature of care provided (if any) and perceptions about additional support that would have been of benefit(Figure 1). This study reports on people who died from "Emphysema/other lung disease".

All available demographic data were used in the analysis. Bivariate Chi-square analyses were used to explore demographic characteristics for caregivers, the deceased and palliative care service use. Bonferroni correction was applied given multiple comparisons. Using the demographic, clinical and service variables that were unlikely to change as a result of the death of someone close, an exploratory logistic regression model was created in PWAS (version 18.0, SPSS Corporation, Carey North Carolina, USA. 2008). This model included seven key factors [17].

### Bias

Strategies to minimise bias included random starting points (choosing CDs and choosing the starting point in CDs), minimising response bias in question design and piloting, and excellent geographic coverage to minimise under-coverage. Non-response is difficult to assess, but weighted and unweighted data were compared to confirm that the sample was representative and, in a sensitivity analysis, unweighted data confirmed the direction and magnitude of findings.

### Ethics, consent and study reporting

The survey received annual South Australian Department of Health Research Ethics Committee approval. Given that this was a community survey in people's homes, verbal consent was acceptable and continued participation accepted as continuing consent. The reporting of this study complies with the STROBE consensus statement on reporting observational studies [18].

## Results

A total of 14, 624 surveys were completed during the period. One in three respondents had someone close to them die an expected death in the five years before the interview (Table 1). Ten percent (1, 504) were 'day-to-day' or 'intermittent' hands-on caregivers for the deceased [19]. Of all deaths, 9.0% were attributed to end-stage lung disease (Table 2). Of these people, 159 (37.1%) accessed specialised palliative care services.

**Table 1:** cross tabulation - respondents to the South Australian Health Omnibus who had someone close to them die from an 'expected' death in the 5 years before responding (n = 1504; weighted data).

**Respondents who had someone close to them die from an expected death in the 5 years before responding who provided care for someone with **...						
		**...end-stage respiratory disease**		**... a diagnosis other than end-stage respiratory disease**		

**Caregivers (respondents)**						

Factors that do not change as caring is relinquished						

	**Factor**	**Fraction^**	**%**	**Fraction^**	**%**	**p value##**

	Gender - male	55/145	37.9	501/1358	36.9	0.806

	Age of respondent - 65+	31/145	21.4	275/1359	20.2	0.745

	Educational attainment - beyond school	70/146	47.9	730/1358	53.8	0.181

	Country of birth - non-English speaking	8/146	5.5	147/1358	10.8	0.044#

	Relationship to the deceased - spouse	20/145	13.8	172/1358	12.7	0.699

Factors that may change as caring is relinquished						

	Household income ≤AU$60, 000####	92/128	71.9	777/1180	65.8	0.170

	Current work status - full or part time*	61/122	50.0	638/1157	55.1	0.278

	Region of residence - metropolitan	86/145	59.3	918/1358	67.6	0.044

	SEIFA index -lowest 60%	73/146	50.0	600/1358	44.2	0.179

Caring characteristics						

	Level of care - day-to-day	65/145	44.8	603/1358	44.4	0.922

	Length of care - ≤ 1 year ###	52/101	51.5	518/919	56.4	0.348

	Had enough support ###	33/128	25.8	326/1152	28.3	0.548

Post-care factors						

	Time since death. ≤ 2 years	84/144	58.3	768/1351	56.8	0.732

	Moving on with life - able to move on	110/146	75.3	1089/1345	81.0	0.104

	Would care again - yes	28/30	93.3	271/290	93.4	0.981

	Sought help for grief or wished they had - yes**	22/69	31.9	215/551	39.0	0.250

**The deceased**						

	Age of the deceased > 65***	63/68	92.6	391/554	70.6	0.000

	Comfortable or very comfortable in the last 2 weeks of life*	18/45	40.0	147/352	41.8	0.821

	Place of death - institution (hospital or hospice)**	50/69	72.5	375/553	67.8	0.434

**Service factors**						

	Palliative care service use - yes	56/146	38.4	841/1358	61.9	0.000

# Fisher's exact test

## Using a Bonferroni correction, a significant p value is 0.0025

### only asked of caregivers

Changes in the denominator reflect years in which the specific question was not asked (* not asked in 2006; ** only asked in 2005, 2006; *** only asked in 2006, 2007) and non-responders where data have not been imputed####

^ Numerators and denominators may also vary +/- 1 due to rounding of weighted data

**Table 2:** Relative contribution of each disease group to expected deaths and uptake of specialist palliative care services within each diagnostic group.

	Percentage (absolute numbers)	
Diagnosis	...of deaths	... who knew specialist palliative care services were utilised
Cancer	77.5 (3683)	52.0 (1917)
Motor neurone disease and other neurodegenerative disorders	3.0 (142)	51.4 (73)
End-stage lung disease	9.0 (428)	37.1 (159)
End-stage cardiac failure	4.9 (235)	28.1 (66)
End-stage hepatic disease	0.8 (39)	35.9 (14)
End-stage renal failure	0.9 (43)	30.2 (13)
AIDS	0.4 (17)	58.8 (10)
Other diagnoses	3.5 (167)	26.3 (44)
Total	100.0 (4754)	48.3 (2296)

2002-03 and 2005-7

Of the 1, 504 active caregivers, 145 cared for people with ESLD (9.6%). In bivariate analyses, there was no difference in the age of caregivers but people dying of ESLD were more likely to be > 65 (ESLD versus all other caregivers, 93% vs. 71%, p < 0.0001; Table 1), have care provided for longer mean times (ESLD - 25 months, 'other diagnoses' 15 months; p < 0.0001) and less likely to access specialised palliative care services (38% vs 62%, p < 0.0001).

Despite less access to specialist palliative care services, domains (Table 3) and sub-domains (Table 4) where additional support would have been useful were similar when comparing caregivers for people with ESLD with other caregivers at the end-of-life.

**Table 3:** Domains where additional support was perceived as being useful by caregivers responding to the South Australian Health Omnibus who had actively provided care (n = 1504) at some time in the five years before responding for someone at the end-of-life.

	**Respondents who had someone close to them die from an expected death in the 5 years before responding who died of **...				
	**...end-stage lung disease** **n = 128**		**...a life-limiting illness other than end-stage respiratory disease** **n = 1152**		**p value**

**Perceived additional support helpful for:**	**n**	**%**	**n**	%	

**physical care, symptom control, medications**	37	28.9	273	23.7	0.192

**information about the disease progression of services available**	23	17.8	200	17.4	0.894

**emotional, spiritual or bereavement support**	26	20.2	293	25.4	0.189

**Finances**	4	3.1	76	6.6	0.175#

**Other additional support**	29	10.2	99	9.9	0.879

# Fisher's Exact Test used

**Table 4:** Perceived additional support would have been helpful comparing people who cared for someone with end-stage lung disease and other caregivers for people at the end of life.

Domains			End-stage lung disease (128)	%	Other diagnoses (1151)	%	p value*
	**Physical care**	Help with physical care	27	12.6	187	16.2	0.163
		
		Help with medications	7	5.4	72	6.3	0.848#
		
		Help with symptom control	13	10.2	115	10.0	0.950
	
	**Information**	Information about what to expect	15	11.7	159	13.8	0.514
		
		Information about services	14	10.9	126	10.9	0.977
	
	**Emotional/spiritual support**	Emotional support for the caregiver	8	6.2	143	12.4	0.043#
		
		Emotional support for the person who died	11	8.6	157	13.6	0.110
		
		Other emotional support	7	5.5	58	5.0	0.831#
		
		Additional spiritual support	1	0.8	49	4.3	0.054#
	
	**Financial support**	Additional financial support	4	3.1	76	6.6	0.175#
	
	**Bereavement Support**	Bereavement support	5	3.9	86	7.5	0.150#

*Using a Bonferroni correction, a significant p value is 0.0045

#Fisher's exact test used.

Given that there was a trend suggesting that people providing care for people with ESLD were *less *likely to identify that additional emotional or spiritual support would be of value, an exploratory binary regression model was constructed. The model controlled for factors which have relevance to the experience of caring: gender, age (as a continuous variable), highest educational achievement, country of birth, the relationship to the deceased (spousal or non-spousal), specialist palliative care service utilisation, and diagnosis. Identifying that additional emotional or spiritual support would have been of value was more likely for younger caregivers (OR 0.977; 95% confidence interval (CI) 0.965 to 0.988; p < 0.0001), people dying of diseases other than ESLD (OR 0.814; 95% CI 0.673 to 0.983; p = 0.033) and females (OR 1.504; 95% CI 1.027 to 2.202; p = 0.036). The Hosmer and Lemeshow goodness-of-fit (p = 0.477) suggested that the model adequately fitted the data, and was confirmed by the Omnibus Tests of Model coefficients (p = 0.000).

## Discussion

This study identifies caregivers for people at the end-of-life at a whole-of-population level, regardless of health service access or utilisation. The study reiterates that caregivers for people at the end of life have perception of significant unmet needs regardless of diagnosis. It follows previous work that explored the characteristics of caregivers for people with ESLD [20] and now compares them with the group who cared for individuals who died of other progressive diseases. Those providing informal care for people with ESLD provided care for significantly longer periods of time than the other diagnostic groups combined, and had much lower levels of support from specialist palliative care services, despite evidence of needs that were of the same magnitude and in similar domains. Importantly, although rates of perceived unmet needs may be seemingly low, the rates did not change for respondents over the five years of the study suggesting that response shift does not account for the rate. The null hypothesis can be rejected - there are specific characteristics and needs that can be identified at a population level.

Chronic obstructive pulmonary disease (COPD) is the major cause of ESLD in our community. People with advanced COPD have been shown to have significant physical and psychosocial care needs at least as intense as people with lung cancer [21-23]. They have a high symptom burden including unrelieved breathlessness and pain [21]. Anxiety and depression rates have been found in up to 90%, and information regarding diagnosis, prognosis and social support is often lacking.

This current study has a younger cohort of (mostly) non-spousal caregivers. In qualitative studies, spousal caregivers of people with advanced COPD have discussed the burden of caring [7,10] and have reported fatigue, loss of concentration and lack of sleep [19]. They have described restrictions on their own lives [6,7,24-26], isolation [6,24,26], anxiety and emotional distress [6,7,24]. They have reported a lack of information about the prognosis and future management of the condition [24], a lack of practical information and information about services [24], a lack of emotional and bereavement support, and a lack of respite services [10]. The review by Caress *et al *concluded there is a dearth of information on the needs of caregivers of people with COPD and interventions that may best support them [13]. This current population-based study supports these findings. The selection of participants for these studies relies largely on contact with health services, whereas the current Omnibus study is across the population irrespective of health service utilization.

Bereaved caregivers of people who died from COPD have indicated that access to support services is limited by a lack of knowledge about, and signposting to available services, a perception among caregivers and, at times, patients that it is embarrassing to ask for help, and a lack of agreement between the patient and the caregiver as to what services should be accessed [10].

Poor rates of service utilisation might relate to the perception that specialist palliative care services are historically for people with cancer or the intensity of the perceived needs are not appreciated by the clinicians as caring is provided over much longer periods of time [27]. Patient-, carer-, referrer- and service-related barriers to specialist palliative care service referral have all been documented despite measured unmet needs [28]. Gysels *et al *have postulated that low access to specialist palliative care services may be due to the nature of breathlessness itself with its surreptitious onset and slow progression; patients' assigning stigma to breathlessness; and the way health professionals often discredit the patients' experiences of breathlessness and merely ascribe the finding to being an expected clinical sign [26].

The length of time for which people experience symptomatic COPD has been noted to be longer than many other end-stage diseases [22]. The progressive limitation of function which occurs over time with intermittent acute worsening of disease (any of which could lead to death) requires a more complex approach to symptom management and caregiver support [29,30]. Frequent episodes of 'coming back from the brink' may give patients a sense of invincibility leading to delayed acceptance of additional support for caregivers. The progressive nature of restrictive or obstructive lung disease over long periods of time may mask people's insight into the fact that death is imminent.

Long periods of caregiving with inadequate support may result from a combination of:

(a) recalibration (response shift) such that the need for service support *appears *to be adequate;

(b) upskilling of caregivers in their roles; and/or

(c) better garnering of resources over time such that the need for service support *is *actually less.

Recalibration can occur where an individual's understanding or perception changes over time through any number of mechanisms and internal processes. This change in perception, or internal standards, is known as 'response shift' which has been rarely documented for caregivers. Studies of caregivers of people with dementia have identified stable caregiver burden and improved psychological and physical well-being despite increased dementia severity suggesting that there may be a caregiver response shift [31].

Another possible parallel explanation is that of upskilling. Given the long period of time for which care is likely to be provided, the upskilling of caregivers (through the gaining of new knowledge, the acquisition of new skills or an increase in confidence in using knowledge and skills) could result. Thus caregivers' ability to care might be enhanced through experiential learning.

Prolonged caring may also enable the garnering of resources such as access to: community resources; increasing support from family and friends; primary care services; or respiratory services. This current dataset cannot delineate between these mechanisms, but does point to the need to define prospectively the contribution that each makes in reducing perceived caregiver burden.

### Strengths of study

This study identifies caregivers from right across the population without gate keeping by clinical staff [3]. This also means that the cohort was not limited to the group most often studied - those who access tertiary services. The study reports caregivers' perceptions of their own needs and hence has no proxy element; this is a crucial basis for considering improvements to support and access to services.

### Study limitations - design

Valid and reliable recall of information is crucial in a retrospective survey. Retrospective assessments may be altered by grief or by the difficulties of recall [32]. Interviews conducted with 1, 271 caregivers 4-12 months after patients' deaths showed good sensitivity and poor specificity concerning hospitalisations, and a moderate degree of agreement with administrative data: agreement increased with educational level and caregiver age [33]. In a recent study of recall of caregiver time using a survey instrument compared with diaries and direct observation, the survey was shown to be an accurate estimate of the amount of informal care provided to patients with dementia [34].

People who had provided informal care were asked to identify the cause of death of the care recipient during the interview. Objective measures of the accuracy of responses are not available.

### Study limitations - sample

Any differential needs for caregivers living in rural or remote communities will not be reflected in these data. Although the data for analysis are weighted to the demographic composition of the whole population, given under-representation of some groups (most notably from culturally and linguistically diverse backgrounds and from Aboriginal and Torres Strait Islander backgrounds) in the initial interviews, it is possible that the responses do not encompass every single issue of note. Needs of people in residential aged care facilities will not be reflected. Given that the weighting is based on ten year age groups, gender and rurality, it is likely that on balance weighting adds rather than detracts from the findings.

### Future research directions that this study informs

The current study reports exploratory whole-of-population data that can help to shape a future research agenda. Ideally, a prospective longitudinal study that directly explores actual unmet needs of caregivers while in the role and after they have relinquished the role is the next step. This will minimise recall bias and may allow for the control of any response shift. It would also be the opportunity to explore the reason for the seemingly low rates of perceived unmet needs expressed by former caregivers in this study. Given the paucity of longitudinal studies of caregivers in general and especially of people with ESLD, there is an urgent need for such studies to be undertaken. Given the reliance of health and social systems on the care provided by family and friends at the end-of-life, it is imperative to know how best to support them. Ultimately unmet needs of caregivers is best reflected in differential outcomes for patients, or in the wellbeing of caregivers while in the role and subsequently.

The strength of these findings suggests that such prospective work could be in the form of a randomised intervention that explores whether the identification and follow up of needs generates better outcomes than identification alone or retrospectively.

Quantitative data need to be complemented with qualitative approaches with caregivers over the time course of caring. This research should identify needs, whether and how they are being met, how they could be better met and how skills and confidence in the role change over time. It should arguably also include how clinicians are assessing caregivers' needs, and whether or how they offer support.

This study builds on earlier work outlining the demographic profile of people providing care at the end-of-life for those with ESLD [20]. These additional data confirm that the domains where additional support would be of use are similar irrespective of the diagnosis despite much lower uptake of specialist palliative care services by people with ESLD.

## Conclusions

Caregiver needs were similar when comparing people providing this care for patients with end-stage lung disease and other diagnoses. The context for this was that there was a much longer period of care in a more elderly population of care recipients. Further work needs to be done to understand why needs do not demonstrate such differentials when this is not the case with other specific diagnoses. This may relate to the length of time for which care is offered given that potentially it would allow sufficient time to mobilise support networks or allow other mechanisms to adjust to the role.

## Competing interests

The authors declare that they have no competing interests.

## Authors' contributions

Conception and design DC, AA, Obtaining funding DC, AA, Acquisition of data DC, AA, Analysis DC, Interpretation of data DC, AA, GC, AW, MF, Drafting of report DC, Critical revision DC, AA, GC, AW, MF. All authors read and approved the final manuscript.

## Funding

The costs for the survey were generously supported by Daw House Hospice Foundation, and salary for Dr Farquhar is provided my Macmillan Cancer Support.

## Pre-publication history

The pre-publication history for this paper can be accessed here:

http://www.biomedcentral.com/1471-2466/11/55/prepub
